# Gene expression profiling of human ovarian tumours

**DOI:** 10.1038/sj.bjc.6603346

**Published:** 2006-09-12

**Authors:** S Biade, M Marinucci, J Schick, D Roberts, G Workman, E H Sage, P J O'Dwyer, V A LiVolsi, S W Johnson

**Affiliations:** 1 Department of Pharmacology, University of Pennsylvania Cancer Center, BRB II/III- Room 1020, 421 Curie Building, Philadelphia, PA, USA; 2 Hope Heart Program, Benaroya Research Institute at Virginia Mason, Seattle, WA, USA

**Keywords:** ovarian cancer, expression profiling, microarray, borderline tumors

## Abstract

There is currently a lack of reliable diagnostic and prognostic markers for ovarian cancer. We established gene expression profiles for 120 human ovarian tumours to identify determinants of histologic subtype, grade and degree of malignancy. Unsupervised cluster analysis of the most variable set of expression data resulted in three major tumour groups. One consisted predominantly of benign tumours, one contained mostly malignant tumours, and one was comprised of a mixture of borderline and malignant tumours. Using two supervised approaches, we identified a set of genes that distinguished the benign, borderline and malignant phenotypes. These algorithms were unable to establish profiles for histologic subtype or grade. To validate these findings, the expression of 21 candidate genes selected from these analyses was measured by quantitative RT–PCR using an independent set of tumour samples. Hierarchical clustering of these data resulted in two major groups, one benign and one malignant, with the borderline tumours interspersed between the two groups. These results indicate that borderline ovarian tumours may be classified as either benign or malignant, and that this classifier could be useful for predicting the clinical course of borderline tumours. Immunohistochemical analysis also demonstrated increased expression of CD24 antigen in malignant versus benign tumour tissue. The data that we have generated will contribute to a growing body of expression data that more accurately define the biologic and clinical characteristics of ovarian cancers.

Ovarian cancer is newly diagnosed in approximately 25 000 women annually in the US and is associated with nearly 15 000 deaths. These grim statistics underscore the need for advances in the development of diagnostic and prognostic markers in addition to new therapeutic approaches. Common epithelial ovarian tumours, which account for the majority of all ovarian neoplasms, are classified as benign, borderline or malignant according to their histology and clinical behaviour (Ozols *et al*, 1997). They are also subdivided into at least seven histological subtypes. Serous tumours account for nearly half of all epithelial ovarian tumours, followed by mucinous, endometrioid and clear cell subtypes, respectively. Of the four major histologic subtypes, serous tumours are associated with the lowest five-year survival rate (20–35%) compared to mucinous (40–60%), endometrioid (40–60%) and clear cell tumours (35–50%). Each subtype may be graded to reflect the extent of architectural and cytoplasmic features of differentiation. The histologic grade is of more prognostic significance than subtype, because a higher five-year survival is associated with well-differentiated tumours (70–80%) in comparison to moderate (30–45%) or poorly differentiated tumours (20%). Although, the histologic subtype, grade and degree of malignancy have significant implications for ovarian cancer patients, there are a number of other important prognostic factors including disease stage, volume of residual disease following debulking surgery, age, performance status and the type of chemotherapy used in treatment (Friedlander, 1998).

Borderline tumours, or tumours of low malignant potential (LMP), have a histologic appearance that lies between that of the benign and malignant phenotypes. These tumours, which account for 10–20% of common epithelial tumours, are characterised by a high proliferation rate without stromal invasion (Trope and Kaern, 1998). The prognosis for women with borderline tumours is excellent; however, a significant percentage (10–15%) of borderline tumours will become malignant and result in fatality. As these tumours are relatively rare and afflict younger women, the issue of sparing fertility is important and affects the clinical management of this disease. Although borderline tumours are officially accepted as a tumour type, considerable debate persists over how to diagnose them accurately and predict their clinical course. Therefore, the identification of reliable molecular markers is necessary to select the cohort of borderline patients that are more likely to develop malignant disease. Women with a higher risk of recurrence could then be treated more aggressively to reduce the mortality rate observed for this group.

Overall, there is a lack of specific markers for classifying the clinical and biologic characteristics of ovarian tumours. Although CA125 has been a useful marker for documenting disease response and progression, it has limited use as a diagnostic tool (Meyer and Rustin, 2000). The advent of expression profiling and proteomic technologies has the benefit of enabling investigators to identify groups of genes and proteins that may be used as classifiers. To date, only a few large studies have used these techniques in an attempt to classify ovarian tumours with respect to subtype, grade and degree of malignancy (Welsh *et al*, 2001; Schwartz *et al*, 2002; Schaner *et al*, 2003; Adib *et al*, 2004). In the present study, we have examined the expression of approximately 7000 genes in a panel of 120 human ovarian tumours that represent various histologic subtypes, grades and degrees of malignancy. An analysis of the data has revealed specific classifiers for some of these phenotypes. These profiles ultimately contribute to a growing consensus of diagnostic and prognostic markers for ovarian cancer and may provide targets for therapeutic intervention.

## MATERIALS AND METHODS

### Tumour specimens

Over 200 frozen human ovarian tumour specimens were obtained from the Cooperative Human Tissue Network. The specimens were obtained from patients through an informed consent protocol and patient identity remained anonymous. We requested tumours representing a representative range of histologic type, grade and degree of malignancy. The pathology information used in this study was obtained from the accompanying pathology report. Total RNA was isolated with Trizol reagent (Invitrogen, Carlsbad, CA, USA) according to the procedure provided by the manufacturer. Each sample was evaluated for integrity by agarose gel electrophoresis; only the samples with intact 18S and 28S ribosomal RNA bands were used. RNA (2–5 *μ*g) was linearly amplified using a modified amplification procedure originally described by Van Gelder *et al* (1990). Only samples that yielded sufficient quantities of amplified RNA (approximately 5 *μ*g) were used for expression profiling.

### Microarray analysis

Fluorescent cDNA probes were prepared based on the Cy3/Cy5 indirect labelling procedures described previously (Schena *et al*, 1995; Hegde *et al*, 2000). As a reference for each sample, we prepared a pooled common reference containing ovarian tumour RNA mixed with Universal Reference RNA (Stratagene Inc., La Jolla, CA, USA). Amplified RNA (5 *μ*g) was combined in a 1.5 ml microcentrifuge tube with 5 *μ*g oligo-dT (Invitrogen) in a final volume of 17.4 *μ*l, incubated at 65°C for 10 min, and placed on ice for 5 min. The cDNA synthesis reaction consisted of a mixture of 6.0 *μ*l 5 × First Strand buffer (Invitrogen), 3.0 *μ*l 0.1 M DTT, 0.6 *μ*l aminoallyl-dUTP/dNTP mix, 1 *μ*l RNasin and 2.0 *μ*l Superscript II reverse transcriptase (200 U/ *μ*l). Samples were incubated at 42°C for 2.0 h. RNA was degraded by adding 10 *μ*l of 1 *N* NaOH and incubating the samples at 65°C for 15 min. Following neutralisation with 10 *μ*l 1 N HCl, the cDNA was precipitated in ethanol, vacuum-dried, and resuspended in 9 *μ*l 0.1 M sodium bicarbonate (pH 9.0). The samples were incubated with Cy5 or Cy3 dye (Amersham, Piscataway, NJ, USA) for 1 h in the dark at room temperature, and the reactions were quenched with the addition of 4.5 *μ*l of 4 M hydroxylamine. Finally, the reaction products were purified using a QIA-quick PCR purification kit, vacuum-dried, and resuspended in 35 *μ*l hybridisation buffer (40% formamide, 5 × SSC, 0.1% *N*-laurylsarcosine).

Approximately 7000 DNA fragments were successfully amplified by PCR using the GF211/GF212 clone sets obtained from Invitrogen (formerly Research Genetics). After purification of the DNA, printing plates were prepared by mixing DNA in an equal volume with DMSO. Glass slide (UltraGap Slides, Corning Inc., Corning, NY, USA) arrays were prepared by printing DNA fragments in duplicate using an Omnigrid Accent arrayer (Genomic Solutions, Ann Harbor, MI, USA). Post-processing of the slides included UV irradiation followed by 1 h incubation at 80°C in a vacuum oven. Prior to use, slides were prehybridised for 2 h at 37°C in 5 × SSC containing 1% (w/v) SDS and 1% (w/v) bovine serum albumin. For hybridisation, samples were incubated at 100°C for 5 min and placed at 42°C for 20 min. Following brief centrifugation, the samples were applied to the microarray slide, which was overlaid with a coverslip and incubated overnight at 42°C in a hybridisation chamber. The slides were washed once with 5 × SSC, 0.1% *N*-laurylsarcosine at 37°C for 5 min, followed by three 5 min washes with 0.1 × SSC at room temperature. The slides were centrifuged to dryness and scanned using an Axon GenePix 4000B scanner (Axon Instruments, Union City, CA, USA).

The raw data were uploaded into Microsoft Excel for analysis. The data were cleaned to eliminate flagged spots. The sample and reference channels were then balanced based on the total fluorescence intensity of the remaining values. As the microarray slides were spotted in duplicate, an average intensity value was calculated for each cDNA element. If the average error between the two spots was 20%, the value was eliminated. Only values were retained in which duplicate intensity measurements were available. The data were normalised by global means normalisation; therefore, the mean of all the averaged values on the array was equal to one. The data were cropped to contain only the cDNA elements that yielded values in at least 80% of the tumour specimens. This final data set was analysed by several statistical algorithms including the Cluster and Tree View programs developed by Eisen *et al* (1998), Prediction Analysis of Microarrays (PAM) program developed by Tibshirani *et al* (2002), and by Student's *t*-test.

### Quantitative RT–PCR

Relative gene expression was measured in each of the cell lines by ‘real time’ quantitative PCR by the use of a LightCycler (Roche Diagnostics Corp., Indianapolis, IN, USA) with SYBR green chemistry. Reaction conditions were optimised for each primer set. Measurements were made in duplicate and normalised based on the average expression of six housekeeping genes (ubiquitin-specific protease 21, ornithine decarboxylase, chaperonin-containing TCP1 subunit 3, glutamate dehydrogenase 1, lactate dehydrogenase A and eukaryotic translation initiation factor 2). Primer sequences and reaction conditions are listed at www.realtimeprimers.org.

### Immunohistochemistry

Formalin-fixed, paraffin-embedded tissues were obtained from the Cooperative Human Tissue Network (University of Pennsylvania, Philadelphia, PA, USA), dewaxed with xylene, and gradually hydrated. Antigen retrieval was achieved by pressure-cooking in citrate buffer (0.01 M, pH 6) for 10 min. The slides were washed and pretreated with Dako Cytomation endogenous peroxidase blocking solution (Hamburg, Germany) for 10 min. Dako Cytomation non-specific antigen blocking protein substrate (Hamburg, Germany) was subsequently applied, and the slides were incubated at room temperature for 15 min. Slides were subsequently incubated with either a 1 : 50 dilution of mouse-anti-CD24 antibody (Santa Cruz – SC7034) or a 1 : 200 dilution of rat-anti-SPARCL1 antibody (SC1, MAST9, Hevin, clone 12–51 supplied by Dr Helene Sage) (Brekken *et al*, 2004) for 1 h at room temperature. After washing and incubation with the appropriate HRP-coupled secondary antibody, detection was carried out using a BD Pharmingen DAB Substarate kit (San Diego, CA, USA) according to the manufacturer's instructions. Slides were counter-stained with haematoxylin.

## RESULTS

### Specimens and data analysis

We established expression profiles for 120 human ovarian tumours obtained from the Cooperative Human Tissue Network. The pathologic characteristics of these tumours are shown in Table 1. The majority of the specimens represented malignant tumours (48%); however, a large percentage of benign (24%) and borderline (28%) tumours was also analysed. With respect to tumour subtype, the cohort consisted primarily of mucinous tumours (42%), followed by serous (23%), clear cell (17%) and endometrioid (9%) subtypes, whereas seven (6%) of the tumours contained a mixture of at least two subtypes. The accompanying pathology reports contained information regarding histologic grade (Table 1). Total RNA was isolated from whole tumour specimens and the integrity of each sample was assessed by agarose gel electrophoresis. We observed that approximately 60% of the tumour specimens yielded intact RNA and only these were subjected to cDNA microarray analysis. Each array contained approximately 7000 genes spotted in duplicate. The resulting data set was cleaned and processed to include only the expression values that (a) differed by 20% between duplicates, and (b) for which data were obtained from at least 80% of the specimens. The data was then batch-corrected and median-normalised. These processing steps resulted in a final data set containing expression values for 5494 cDNA elements.

### Unsupervised classification

**Figure 1:**
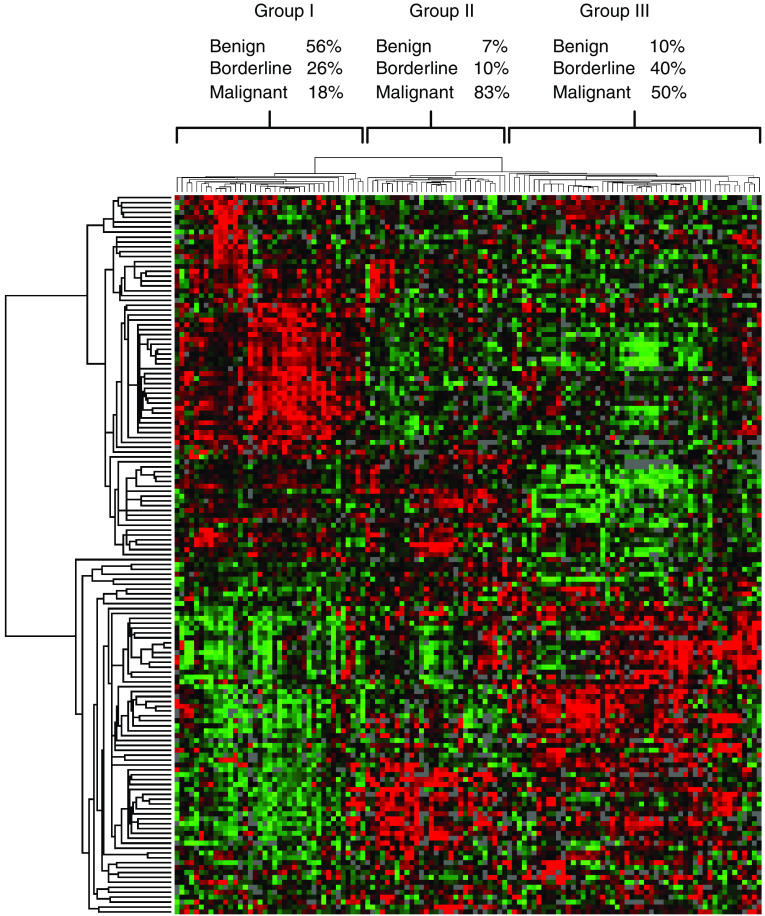
Unsupervised hierarchical clustering of benign, borderline and malignant human ovarian tumours. The most variable portion of the data set (147 genes) was analysed and visualised by the Cluster and TreeView programs developed by Eisen *et al* (1998). The relative percentage of each tumour type within each major cluster is shown. Red, green and black pixels indicate relatively high, low and neutral expression, respectively.

Hierarchical clustering was applied to the most variable portion of the data to determine whether the phenotypic features of ovarian tumours could be readily distinguished (Figure 1). Three major groups resulted from this analysis and the relative percentages of the benign, borderline and malignant tumours present in each group are shown. Group I contained predominantly benign and borderline tumours (56 and 26%, respectively), while few malignant tumours were present. Group II contained a high percentage of malignant tumours (83%), but few benign or borderline tumours were present. Group III was heterogeneous and contained similar percentages of borderline (40%) and malignant tumours (50%) and relatively few benign tumours (10%). The genes that were increased in expression and associated with groups I and II relative to the other tumour samples are listed in Table 2. This unsupervised clustering algorithm did not clearly distinguish the tumours based on subtype or histologic grade. However, group II contained mostly serous and endometrioid tumours, whereas group III contained a higher percentage of clear cell and mucinous tumours. The average grade of the tumours in group II was moderate to poor, whereas the average grade of the tumours in group III was moderate. The entire data set and detailed dendograms for Figure 1 are available in the (Supplementary Figures S1 and S2).

### Supervised classification

The PAM program was used to develop a classifier for the benign, borderline and malignant phenotypes (Tibshirani *et al*, 2002). This algorithm represents a modification of the nearest-centroid method and identifies genes that best characterise each class. From this analysis, 25 genes were found to be differentially expressed among the three classes (Table 3). The majority of the genes (88%) were upregulated in the benign tumours relative to the borderline and malignant tumours, whereas only three genes (12%) were upregulated in the malignant tumours relative to the other tumour types. The mean expression value obtained for each gene in the borderline class was either equal to or between that of the benign and malignant tumour types. Comparison of the genes upregulated in benign tumours identified by PAM with that of the ‘benign’ cluster listed in Table 2 revealed a common set of nine genes. In addition to PAM, we used a Student's *t*-test to identify genes that were differentially expressed between benign and malignant tumours. A set of data was extracted containing genes that differed in mean expression by two-fold or higher between the benign and malignant tumour types with a P-value 0.05 (Supplementary Table SI). A total of 26 genes was selected, and 15 of the 25 genes identified by PAM were contained in this list. For each gene, the mean expression value for the LMP group was equal to or between that of the benign and/or malignant group.

### Validation by RT–PCR

**Figure 2:**
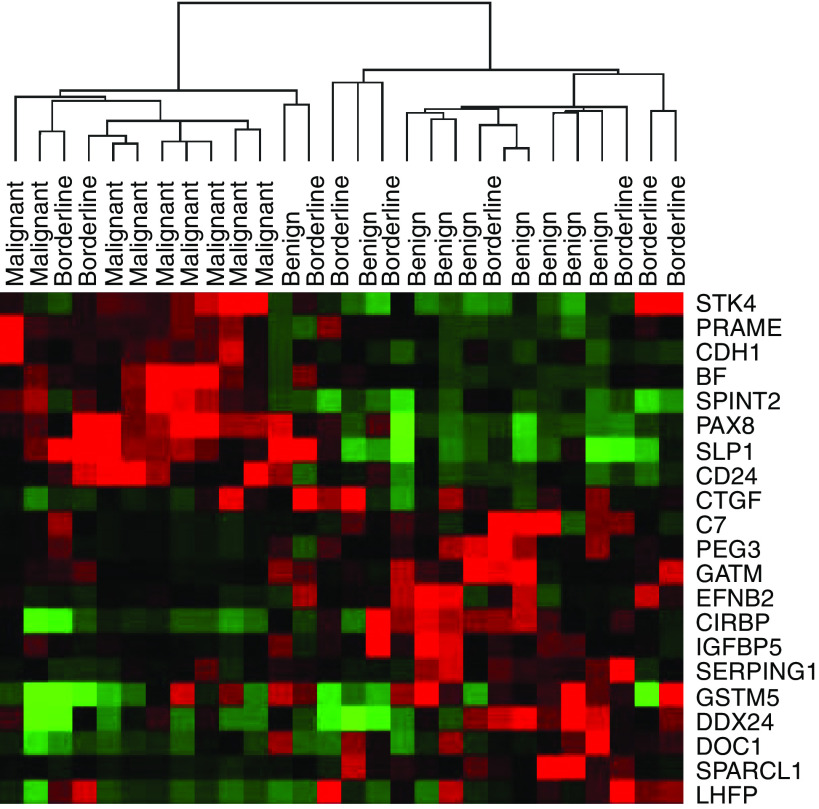
Cluster analysis of quantitative RT-PCR data obtained for 21 genes associated with the benign, borderline and malignant phenotypes. Red, green and black pixels indicate relatively high, low and neutral expression, respectively.

From the unsupervised and supervised analyses described above, we selected 21 genes for validation by quantitative ‘real time’ PCR using an independent set of tumour RNA samples representing nine benign, nine borderline and 10 malignant ovarian tumours. The expression of these genes was measured relative to a set of six housekeeping genes. For quantitation, a standard curve was generated using serial dilutions of the same RNA that was used as a reference in the microarray experiments. Of the genes that were analysed, the mean expression value for each gene was consistent with the trend observed in the microarray data with the mean of the borderline tumours positioned in the middle. There was a significant difference (*P*<0.05) between the means of the benign and malignant groups for 18 of the 21 genes as determined by ANOVA (Supplementary Table SII). The full set of RT–PCR data is available in the Web Supplement. To demonstrate the difference between the phenotypes visually, we applied hierarchical clustering to the RT–PCR data (Figure 2). The resulting dendogram consisted of two major groups: one containing the benign tumours and the other consisting of the malignant tumours. The expression profile of one of the benign tumours was associated with that of the malignant group. The borderline tumours were interspersed among the two clusters; however, the majority (78%) was associated with the benign tumour class.

### Validation by immunohistochemistry

**Figure 3:**
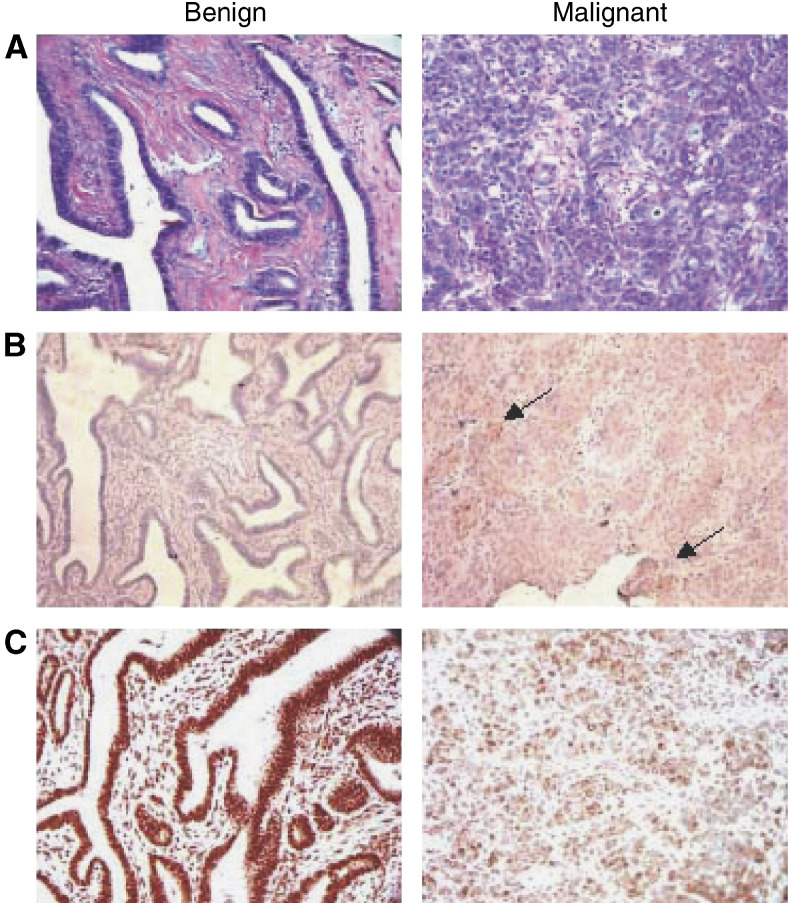
Validation of differentially expressed genes by immunohistochemistry. (**A**) Representative H&E staining of a poorly differentiated serous papillary carcinoma and a benign endometrioid adenomyofibroma. (**B**) Staining of the same tumours with anti-CD24 antibody. The benign tumour showed weak staining whereas the malignant tumour showed moderate to strong signal. Areas of intense staining indicated by arrows. (**C**) Staining of both tumours with anti-SPARCL1 antibody. The benign endometrioid adenomyofibroma exhibited moderate to intense staining while the serous papillary carcinoma showed moderate staining with more prominent areas.

To validate differential expression of two candidate markers at the protein level, we performed immunohistochemistry to detect CD24 antigen and SPARCL-1 protein in a pair of slides representing a benign and malignant tumour (Figure 3). Weak to no signal was detected in a slide representing benign endometrioid adenomyofibroma when stained with CD24 antibody, while in contrast, serous papillary carcinoma exhibited moderate to strong CD24 staining in the nucleus and cytoplasm. With respect to SPARCL1, strong staining was observed in both tumour cells and stroma of the endometrioid adenomyofibroma. Although somewhat weaker, a similar staining pattern was observed in the malignant tumour, with staining of both tumour and stroma.

### Classification by tumour subtype and grade

Tumour subtype and grade are phenotypes designated by microscopic analysis. Therefore, we used supervised algorithms to determine whether molecular classifiers for these histologic features could be extracted from the microarray data. Prediction analysis of microarays analysis was applied to the expression data obtained for 85 borderline and malignant tumour specimens for which subtype information was available. The data set was split randomly into two groups: a training set consisting of data for 63 tumour specimens (14 serous, 26 mucinous, 15 clear cell and eight endometrioid) and a test set which contained data for 22 specimens. With respect to subtype, the relative number of specimens in the test set was proportional to that of the training set. Using a significance threshold of 2.1, PAM generated a set of 149 genes that distinguish the four major subtypes (Supplementary Table SIII). This classifier correctly categorised each of the nine mucinous tumours in the test set. However, none of the clear cell or endometrioid tumours was correctly identified, and only 40% (two of five) of the serous tumours were predicted correctly. A similar approach was used to develop a classifier for histologic grade. To facilitate the analysis, we combined well- and well-to-moderately differentiated tumours with moderately differentiated tumours to form one group (moderate group). Likewise, poor-to-moderately differentiated tumours were combined with poorly differentiated tumours to form a second group (poor group). The data set was then randomly divided into a training set (38 samples) and a test set (13 samples). Using a significance threshold of 1.7, PAM analysis produced a set of 15 genes to serve as a classifier (Supplementary Table SIV). Prediction analysis of the test set revealed that only one of six moderate tumours was classified correctly, whereas all of the seven poorly differentiated tumours were identified correctly.

## DISCUSSION

Expression profiling has proven to be a powerful tool for tumour classification (Dubba-Subramanya *et al*, 2003). Following the pioneering study of Alizadeh *et al* (2000), which established a classifier for B-cell lymphomas, a number of data sets have been generated that contain expression signatures for various biologic and clinical tumour phenotypes. Despite these advances, however, microarray studies are fraught with potential pitfalls that, if not carefully considered, can lead to erroneous conclusions (Simon, 2003). These issues include experimental design, sample size, data analysis and validation using an independent set of samples. In the present study, we established gene expression profiles for 120 human ovarian tumours to identify determinants of tumour subtype, grade and degree of malignancy. We employed both unsupervised and supervised algorithms to generate a set of candidate genes that could serve as a classifier for tumour malignancy. As an initial step towards validating candidate genes as tumour markers, we measured gene expression by quantitative RT–PCR using RNA isolated from an independent set of tumour specimens. We used this validation strategy as an alternative to establishing a test set from the microarray data, as it represents a more accurate method for measuring gene expression. The results indicate that, collectively, these genes are useful markers for the classification of ovarian tumours with respect to degree of malignancy.

To gain insight into the putative function of some of the genes in our classifier, we examined the literature for reports of their involvement in neoplasia. One would predict that if a gene is lost or down regulated in a malignant tumour, its over expression may confer reduced proliferation, differentiation or a non-metastatic phenotype. Conversely, genes that are up regulated in malignant tumours may be more likely to confer a more aggressive, metastatic phenotype. For example, we found that connective tissue growth factor (CTGF) is highly expressed in benign tumours relative to malignant tumours. Chang *et al* (2004) showed that reduced expression of CTGF was associated with advanced-stage disease, lymph node metastasis and shorter median survival in lung adenocarcinoma. Furthermore, invasive and metastatic activity was lower in tumour cells that were engineered to overexpress CTGF. Although no specific functional data are available, Mok *et al* (1994) identified DOC1 (downregulated in ovarian cancer) using a DNA-fingerprinting approach to find genes differentially expressed between ovarian cancer cells and normal ovarian epithelial cells.

SPARC-like 1 (SPARCL1, MAST9, hevin, SC-1) is a member of the SPARC family (Claeskens *et al*, 2001). This gene was originally shown to be downregulated in human non-small cell lung cancer and subsequent reports indicated that downregulation of SPARCL1 also occurs in prostate and colon carcinomas (Bendik *et al*, 1998; Isler *et al*, 2001). This suggests that SPARCL1 inactivation is a frequent event in tumours of epithelial origin. Consistent with our findings in ovarian cancer, Oka *et al* (2001) used differential display to show increased expression of complement component 7 (C7) in normal vs. malignant oesophageal specimens. *In situ* hybridization confirmed the localisation of C7 mRNA in normal oesophageal epithelial cells and its disappearance in tumour cells. Two other genes that have been studied functionally with respect to growth suppression are ephrin-B2 and cold-inducible RNA-binding protein (Nishiyama *et al*, 1997; Liu *et al*, 2004). The latter has been shown to be downregulated in endometrial cancer in comparison to normal endometrium and tissue representing endometrial hyperplasia (Hamid *et al*, 2003). Likewise, we found that ephrin-B2 and CIRBP were expressed at lower levels in malignant tumours compared to benign tumours. An effect of cold-inducible RNA binding protein on reducing cell doubling time was confirmed in our laboratory following transfection of the full-length cDNA into ovarian cancer cells (data not shown).

We discovered that fewer genes were consistently upregulated in malignant tumours relative to benign tumours. Differential expression of these same genes has been observed by other investigators in comparisons of normal vs malignant ovary (Welsh *et al*, 2001; Adib *et al*, 2004). Although the experimental designs do not enable a direct comparison with our results, we may make inferences based on differential expression observed between normal and malignant tumours to that of benign vs malignant tumours. Welsh *et al* (2001) measured gene expression in a set of 27 serous papillary adenocarcinomas of the ovary and three normal ovarian tissue samples. Of the 30 genes that were considered most significantly different between the two groups, three (CD24, PAX8, SPINT2) were among the upregulated genes resulting from our analysis. Adib *et al* (2004) established expression profiles for tissue specimens representing four normal ovary, six primary tumour and six corresponding tumour metastases. Consistent with our results, they observed upregulation of B-factor (properdin), CD24 antigen, E-cadherin, opioid-binding protein, preferentially expressed antigen in melanoma (PRAME) and antileukoprotease (SLP1) in primary tumours and metastases. Some of these genes may be specific to ovarian tumours, whereas others may be expressed in other tumour types relative to corresponding normal tissue. For example, CD24 is a sialoglycoprotein that is anchored to the cell surface by a glycosyl phosphatidylinositol (GPI) linkage and normally expressed in a variety of haematopoietic cells. Kristiansen *et al* (2002, 2003, 2004) have reported that increased CD24 expression is associated with poor prognosis in breast, prostate, and ovarian cancer. PRAME encodes a 509 amino-acid protein that is capable of eliciting a T-cell response. Expression of this antigen is primarily restricted to the testis, but has been shown to be expressed in a variety of solid and haematologic tumours (Matsushita *et al*, 2003). Increased expression of antileukoprotease (SLP1) has also been observed in a variety of tumour types including ovarian cancer (Shigemasa *et al*, 2001). Devoogdt *et al* (2003) demonstrated that transfection of the human SLP1 cDNA into Lewis lung carcinoma cells resulted in increased tumourigenicity and lung-colonising potential. In contrast, however, the protease inhibitor SPINT2 was shown to reduce the metastatic potential of ovarian cancer cells (Suzuki *et al*, 2003). This result highlights the complexity of defining the specific function of genes that ultimately control tumour growth and metastasis. Finally, there is evidence to suggest that E-cadherin is associated with the malignant phenotype. In a study by Sundfeldt *et al* (2001), the levels of soluble E-cadherin were significantly higher in cystic fluid from cystadenocarcinomas and borderline tumours compared to cystic fluid from cystadenomas. Collectively, the association between many of the upregulated genes that we identified and expression/function in other published studies of tumourigenesis suggest that they may contribute functionally to the metastatic ovarian cancer phenotype. Whether or not they represent useful therapeutic targets will require further study.

Recently, Shih and Kurman (2004) proposed a model to describe the pathogenesis of ovarian cancer based on morphologic and molecular data. Tumours were classified into two major groups (types I and II) with respect to potential tumourigenic pathway. In this model, type I tumours consist of low-grade neoplasms that undergo stepwise transformation from benign and borderline tumours into malignant carcinomas. This group includes low grade serous tumours in addition to tumours representing the other major subtypes. Type II tumours consist primarily of high-grade serous carcinomas which are inherently more aggressive and evolve *de novo* from the ovarian surface epithelium or inclusion cysts. These tumours metastasise rapidly and are associated with a lower five-year survival in comparison to type I tumours. Consistent with this idea, our microarray data have also defined two major malignant tumour classes: One (Figure 1 – group II) that contained a high percentage of high grade malignant tumours, and the other (Figure 1 – group III) that consisted primarily of a mixture of borderline and malignant tumours of moderate grade. Group II contained a mixture of endometrioid and serous tumours, whereas group III represents a mixture of tumour subtypes. It would be of interest to determine whether these groups arise from the type I and type II tumorigenic pathways proposed.

A debate exists regarding which factors are of prognostic significance in borderline ovarian cancer patients. A large retrospective study of 370 patients was conducted by Kaern *et al* (1993) at the Norwegian Radium Hospital. Univariate analysis of this cohort revealed several markers of prognostic significance including FIGO stage, presence of residual tumour, surgical procedure, tumour growth on the ovarian surface and presence of pseudomyxoma peritonei. A more recent study by Trimble *et al* (2002) found FIGO stage to be associated with survival in 2818 women with borderline ovarian tumours. Similarly, Sherman *et al* (2004) reported reduced 10 year survival in distant stage vs localised disease in a group of approximately 4500 borderline ovarian cancer patients. To date, there have been few comprehensive analyses of molecular determinants of prognosis. Our expression profiling results indicate that as a group, borderline tumours are heterogeneous and exhibit features of both benign and malignant tumours. However, there were no specific markers that distinguished borderline tumours as an independent group. These results suggest that it may be necessary to classify borderline tumours into two major groups: one that represents tumours with a benign clinical phenotype (LMP benign) and one that represents tumours that are either malignant or have a higher propensity of becoming malignant (LMP malignant). This concept has been supported by other studies of ovarian tumour pathology (Seidman and Kurman, 2003). However, further analysis will need to be carried out with a larger set of borderline tumours with recurrence and survival data.

From our data, molecular determinants that define ovarian tumour subtypes and grades were not as easily identified as those that distinguish degrees of malignancy. Unsupervised clustering did not group tumours based on subtype or grade, and we were unable to validate completely the classifiers generated by supervised methods. This could be due to a variety of biological and technical reasons. The relative magnitude of differential expression among the genes that distinguish these phenotypes may be relatively low. Therefore, the noise associated with the microarray data could interfere with the identification of such patterns. As we used RNA isolated from whole tumour specimens, the inclusion of stromal, immunologic, and other cell types could serve to dilute relevant gene expression signals. The use of microdissected tissues could resolve this problem. Also, we utilised an array containing 7000 cDNA elements. Given that this does not fully represent the entire human transcriptome, we may have missed relevant genes. Other groups have identified markers that are associated with specific ovarian tumour subtypes. For example, Schaner *et al* (2003), using cDNA microarray analysis, identified a set of genes that are differentially expressed in clear cell tumours compared to other ovarian subtypes. Of the 49 genes listed in this report, 27 of the genes were contained in our data set; however, only one (ESR1) was significantly differentially expressed (*P*<0.05) in our set of clear cell tumours. With respect to tumour grade, we identified only one gene (GSTM2) that was significantly different among 10 genes that were present in both data sets. In the study by Schwartz *et al* (2002), a signature for clear cell tumours was also provided. Comparison of our PAM results with this report indicated that only nidogen 2 was shared between the two data sets. These results underscore the importance of validating microarray results with an independent set of samples.

In conclusion, the results of our study demonstrate that expression profiling is a useful method for classifying ovarian tumour phenotypes. Although a clear expression pattern did not emerge that could classify the individual ovarian tumours by histologic subtype and grade, a set of genes was discovered that could distinguish benign from malignant phenotypes. It is important to consider that the gene expression data were collected from whole tumour tissue rather than microdissected material. In some instances, differential gene expression may represent increased or decreased levels of nontumour mRNA contributed by stromal cells in the whole tissue sample. Upon examination of a selected set of tumours, we found that the malignant specimens consisted of 78% tumours cells versus 45% in the benign. It is unlikely that this difference in stromal content can account for the relatively large differences observed in gene expression between the benign and malignant tumours. Moreover, we were able to demonstrate differential expression of CD24 in benign vs malignant tumours by immunohistochemistry; however, we could not definitively show significant differential expression of SPARCL1, as significant positive staining was observed in stromal cells from both the benign and malignant tumours. These findings underscore the necessity to validate gene expression data by immunohistochemistry or other methods in a larger set of tumour specimens. This will be required to establish definitively the usefulness of these determinants as diagnostic/prognostic markers and to address sensitivity and specificity parameters of such an assay. Overall, the incorporation of our results into those of other investigators will be an important step in identifying a consensus set of markers to improve both the diagnosis and management of ovarian cancer.

## Figures and Tables

**Table 1:** Pathologic characteristics of the ovarian tumours used in this study

**Degree malignancy**	**Subtype**	**Number**
Benign	Mucinous	15
	Serous	9
	Clear cell	0
	Endometrioid	1
	Mixed/other	4
	Total	29 (24%)
		
Borderline	Mucinous	30
	Serous	3
	Clear cell	0
	Endometrioid	1
	Mixed/other	0
	Total	34 (28%)
		
Malignant	Mucinous	5
	Serous	16
	Clear Cell	20
	Endometrioid	10
	Mixed	6
	Total	57 (48%)
		
**Tumour grade**	**Number**	
Well (I)	9 (17%)	
Well-moderate	4 (8%)	
Moderate (II)	10 (19%)	
Moderate-poor	8 (15%)	
Poor (III)	21 (40%)	

**Table 2:** Genes upregulated and associated with group I (benign cluster) and group II (Malignant cluster) as determined by hierarchical clustering

**Accession no.**	**Symbol**	**Genes upregulated in benign tumours**
AI870821	SOX10	SRY (sex determining region Y)-box 10
AA664101	ALDH1A1	Aldehyde dehydrogenase 1 family, member A1
AA477400	TPM2	Tropomyosin 2 (beta)
H99676	COL6A1	Collagen, type VI, alpha 1
R62603	COL6A3	Collagen, type VI, alpha 3
AA683077	MAPK1	Mitogen-activated protein kinase 1
H95960	SPARC	Secreted protein, acidic, rich in cysteine (osteonectin)
AA490172	COL1A2	Collagen, type I, alpha 2
N62586	ERCC5	Excision repair cross-complementing rodent repair deficiency, complementation group 5
AA071473	MATN2	Matrilin 2
R61229	GATM	Gycine amidinotransferase (L-arginine:glycine amidinotransferase)
AA465216	D8S2298E	Reproduction 8
AA682423	MAOB	Monoamine oxidase B
T68892	SFRP1	Secreted frizzled-related protein 1
T90767	CCNT1	Cyclin T1
AA490471	SPARCL1	SPARC-like 1 (mast9, hevin, SC-1)
AA778198	PBX3	Pre-B-cell leukemia transcription factor 3
N58145	LHFP	Lipoma HMGIC fusion partner
W15267	LRP6	Low-density lipoprotein receptor-related protein 6
H08561	IGFBP5	Insulin-like growth factor binding protein 5
AA598794	CTGF	Connective tissue growth factor
T62048	CIS	Complement component 1, s
		
**Accession no.**		**Genes upregulated in malignant tumours**
R38201	OPCML	Opioid-binding protein/cell adhesion molecule-like
H97778	CDH6	Cadherin 6, type 2, K-cadherin (fetal kidney)
AI924523	NAP1	Pronapsin A
AA419229	MGC29643	Hypothetical protein MGC299643
R93509	STK4	Serine/threonine kinase 4
AA434373	ELF3	E74-like factor 3 (ets domain transcription factor, epithelial-specific)
H59916	CD24	CD24 antigen
H97778	CDH1	Cadherin 1, type 1, E-cadherin (epithelial)
AW050484	TSPAN1	Tetraspan 1
H13688	GALNT3	UDP-*N*-acetyl-alpha-D-galactosamine:polypeptide *N*-acetylgalactosaminyltransferase 3
AA022949	GFG18	Fibroblast growth factor 18
AA405767	PAX8	Paired box gene 8
AA911661	HOXB2	Homeo box B2
AA683520	SLP1	Secretory leukocyte protease inhibitor (antileukoprotease)
AA401441	BF	B-factor, properdin
W72393	RAMP3	Receptor (calcitonin) activity modifying protein 3 (RAMP3)

**Table 3:** Identification of genes that distinguish among benign, borderline and malignant ovarian tumours by prediction analysis of microarrays

			**PAM Score**		
**Accession no.**	**Symbol**	**Gene**	**Benign**	**Borderline**	**Malignant**
H08561	IGFBP5	Insulin-like growth factor binding protein 5	0.2760	0	−0.0547
AA977242	CIRBP	Cold-inducible RNA-binding protein	0.2298	0	0
AA459941	PEG3	Paternally expressed 3	0.2251	0	0
AA001614	INSR	Insulin receptor	0.1515	0	0
AA424584	LTBP2	Latent transforming growth factor beta binding protein 2	0.1270	0	0
AA448277	FOXO1A	Forkhead box O1A (rhabdomyosarcoma)	0.1233	0	0
AA481438	SERPING1	Serine (or cysteine) proteinase inhibitor, clade G (C1 inhibitor), member 1	0.1197	0	0
AA700832	RBP1	Retinol binding protein 1, cellular	0.1105	0	0
AA481026	SMARCA2	SWI/SNF related, matrix associated, actin dependent regulator of chromatin, subfamily a, member 2	0.1064	0	0
AA156802	LAMB2	Laminin, beta 2 (laminin S)	0.1022	0	0
AA056232	GSTM5	Glutathione S-transferase M5	0.0977	0	0
AA679454	STAR	Steroidogenic acute regulatory protein	0.0815	0	0
AA682423	MAOB	Monoamine oxidase B	0.0663	0	−0.0675
AA398366	SH3GL1	SH3-domain GRB2-like 1	0.0514	0	0
N78902	LEPR	Leptin receptor	0.0481	0	0
AA405767	PAX8	Paired box gene 8	0	0	0.0471
AA478553	DCT	Dopachrome tautomerase	0.0447	0	0
AA045735	—	Transcribed sequence with moderate similarity to protein sp:P39195	0.0345	0	0
AA460833	PGCP	Plasma glutamate carboxypeptidase	0.0147	0	0
AA490471	SPARCL1	SPARC-like 1 (mast9, hevin, SC-1)	0	0	−0.0093
W15267	LRP6	Low density lipoprotein receptor-related protein 6	0	0	−0.008
AA464856	ID4	Inhibitor of DNA binding 4, dominant negative helix-loop-helix protein	0.0036	0	0
AI934925	SLC23A1	Solute carrier family 23 (nucleobase transporters), member 1	0	0	0.0013
R38201	OPCML	Opioid-binding protein/cell adhesion molecule-like	0	0	0.0012
H99676	COL6A1	Collagen, type VI, alpha 1	0.0005	0	0

The score derived from this analysis using a significance threshold of 3.1 is shown.
